# Bibliometric Analysis and Visualization of Clinical Trials on the Therapeutic Potential of Essential Oils (1967-2024)

**DOI:** 10.7759/cureus.57430

**Published:** 2024-04-01

**Authors:** Namrata Dagli, Mainul Haque, Santosh Kumar

**Affiliations:** 1 Dentistry, Karnavati Scientific Research Center, Karnavati School of Dentistry, Karnavati University, Gandhinagar, IND; 2 Therapeutics, Karnavati Scientific Research Center, Karnavati School of Dentistry, Karnavati University, Gandhinagar, IND; 3 Pharmacology and Therapeutics, National Defence University of Malaysia, Kuala Lumpur, MYS; 4 Periodontology and Implantology, Karnavati Scientific Research Center, Karnavati School of Dentistry, Karnavati University, Gandhinagar, IND

**Keywords:** minor tranquillizer, aetheroleum, ethereal oils, scientometric analysis, mental health, dental health, anxiolytic, antimicrobial, network analysis, bibliometric

## Abstract

Essential oils, aromatic compounds extracted from various parts of plants, have garnered significant attention in recent years due to their diverse therapeutic properties and potential applications in healthcare. This analysis delves into the publication trends, productivity patterns, most relevant contributors, coauthorship networks, most frequently used keywords, and their co-occurrence, topic trends, thematic evolution, and collaboration between various countries in clinical trials exploring the therapeutic potential of essential oils. Six hundred sixty-one clinical trials were selected from the PubMed database for analysis, authored by 2959 authors, and published across 359 sources. The analysis identified Horrobin DF as the most contributing author based on the number of published clinical trials, followed by Kasper S, McGuire JA, and Schlafke S. Lotka's law underscores the distribution of authors' productivity, revealing a small number of highly productive authors. Coauthorship analysis identifies significant collaborations among authors and institutions, with prominent contributors like Siegfried Kasper and institutions like Shiraz University of Medical Sciences. Furthermore, the analysis highlights leading journals like *Complementary Therapies in Clinical Practice* and the *Journal of Alternative and Complementary Medicine*. Using keyword clustering, connections between various subjects and their chronological presence are uncovered, offering insights into the changing research landscape. The thematic examination exposes changes in research emphasis over time, progressing from fundamental studies on essential oil components to broader utilization and focused inquiries into oils and therapeutic domains. Analysis of the countries of corresponding authors revealed that Iran has the highest number of multiple-country publications. Moreover, international collaboration trends have been unveiled. Together, these analyses furnish holistic understandings of keyword relationships, thematic shifts, and global partnerships in essential oil research, presenting valuable perspectives on trends and focal points within this domain.

## Introduction and background

In recent years, there has been a burgeoning interest in exploring natural remedies as alternatives or complements to conventional pharmacological interventions. Among these, essential oils have gained significant attention due to their diverse therapeutic properties and historical use in traditional medicine systems. Essential oils are concentrated hydrophobic liquids containing volatile aroma compounds from plants. These oils are extracted from various parts of plants, such as leaves, flowers, stems, roots, bark, or fruits, through distillation, expression, or solvent extraction. Essential oils are known for their characteristic fragrances and are used in aromatherapy, perfumery, cosmetics, and traditional medicine practices [1,2].

The composition of essential oils can vary widely depending on the plant species, growing conditions, and extraction methods used. They typically consist of complex mixtures of volatile organic compounds, including terpenes, phenylpropanoids, phenols, alcohols, ethers, aldehydes, and ketones [3,4]. Each essential oil has a unique chemical profile that contributes to its specific aroma and potential therapeutic properties.

Essential oils have been used for centuries in various cultures worldwide for their purported medicinal properties. They are believed to possess multiple therapeutic effects, including antimicrobial, anti-inflammatory, analgesic, antispasmodic, and antioxidant activities [5-7]. As a result, essential oils have gained increasing attention in modern scientific research and are being explored for their potential applications in healthcare, wellness, and skincare. Their unique chemical composition and biological activities make them promising candidates for developing novel therapeutic agents.

Bibliometric analysis of the therapeutic potential of essential oils is crucial due to the vast and complex nature of research in this field, which spans a wide array of therapeutic applications. With the ever-increasing volume of published scientific literature, it becomes challenging to review and analyze the existing body of knowledge systematically. Bibliometric analysis, a quantitative method widely used in scientific research, offers a comprehensive approach to assessing the scientific landscape, trends, and patterns within a particular field. By systematically analyzing key research themes, influential authors, leading sources, leading research groups, and collaborative networks, this bibliometric analysis aims to shed light on the current state of knowledge, leading contributors, collaboration patterns, research gaps, and future research directions in the field of essential oil research.

## Review

Material and methods

Data Collection

An online literature search was conducted in PubMed on March 11, 2024, to ensure a broad and multidisciplinary coverage of literature related to the therapeutic applications of essential oils in various fields. Boolean search queries were carefully formulated to capture relevant articles. The search query used was - (Essential-oils AND dent*) OR (Essential-oils AND therapy*) OR (Essential-oils AND Antiviral) OR (Essential-oils AND Antimicrobial) OR (Essential-oils AND Antifungal) OR (Essential-oils AND Anxiolytic) OR (Essential oils AND Medicin*) OR (Essential oils AND health) NOT (Essential oils AND Cosmetic*). The clinical trials, published in English, focused on the therapeutic use of essential oils across diverse disciplines, were included in this analysis. The filters in the PubMed database were applied to filter out reviews, editorials, observational studies, books, and other documents. Metadata, including title, authorship, publication year, journal, and abstract, were extracted from all the selected articles and added to a separate text file. The flow chart of the study selection process was generated according to the Preferred Reporting Items for Systematic Reviews and Meta-Analyses guidelines [8].

Data Analysis

Bibliometric software tools, VOSviewer [9] and Biblioshiny [10] were employed to analyze and visualize bibliographic data. These tools facilitate the creation of coauthorship networks, collaboration maps, thematic clusters, identification of leading contributors, analysis of topic trends, and graphical representation of research trends. The Biorender App and Microsoft Excel were also used to visualize data. By implementing these rigorous methods, this bibliometric analysis offers a robust and systematic exploration of the existing literature on the therapeutic applications of essential oils, contributing to a deeper understanding of research trends, collaborations, themes, and influential contributors in this evolving field. The study adhered to ethical guidelines, respected copyright laws in using published materials, and proper attribution to the original authors and sources.

Results

**Figure 1:**
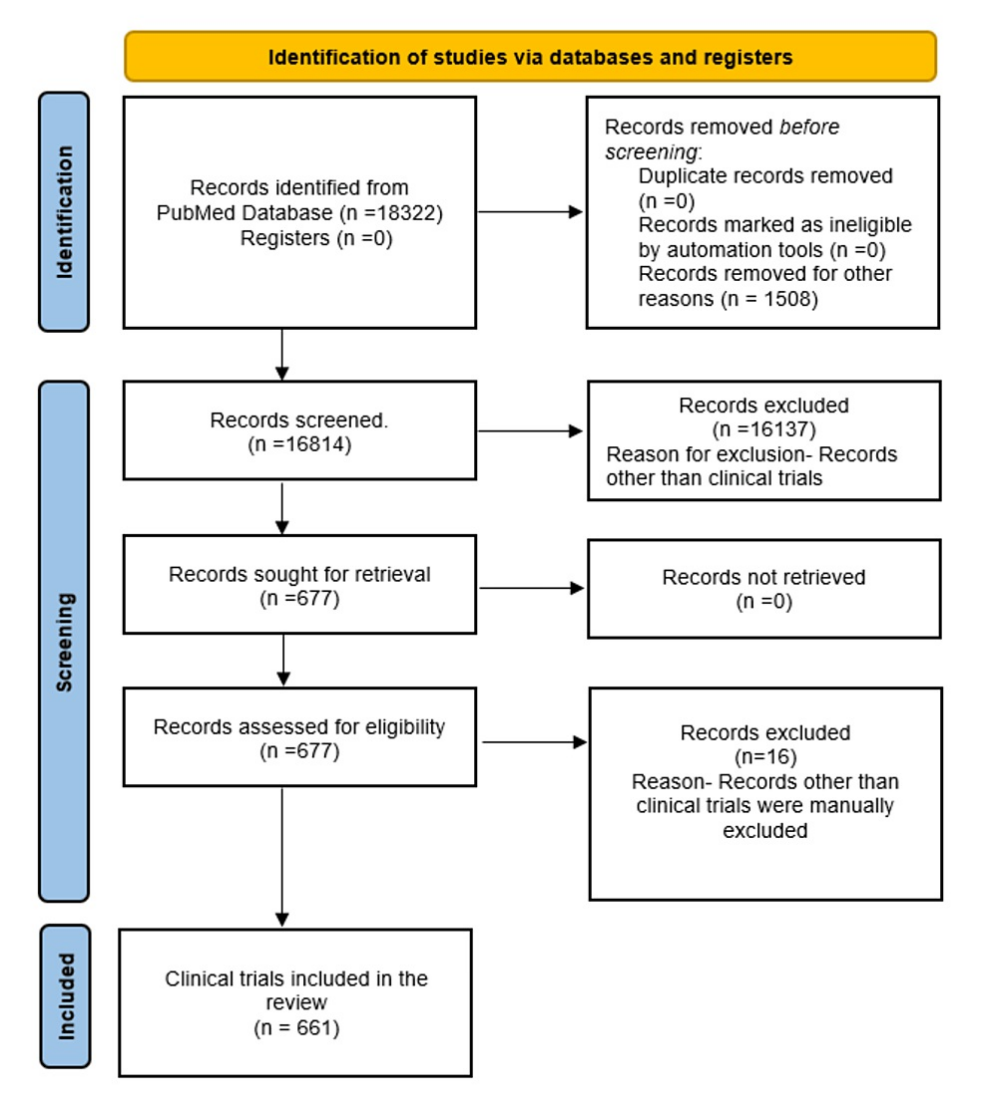
The flow chart depicts the process of selecting studies Credit: Namrata Dagli

Out of a total of 18322 results that appeared in the PubMed database, 16814 met the criteria of the English filter. This set contained 2185 reviews, 159 case reports, 34 editorials, 19 books, and other documents. Additionally, there were 677 clinical trials spanning different phases, with five studies in phase I, 9 in phase II, 4 in phase III, and 1 in phase IV (Figure 1). No duplicate publications or twin studies were found in the PubMed database. After further refinement, which involved eliminating comments, reviews on clinical trials, protocols of reviews, and retracted clinical trials manually, a total of 661 articles were selected for inclusion in the analysis. The clinical trials were authored by 2959 individuals and published in 359 distinct sources. Among these authors, 20 contributed single-authored documents, while international coauthorship stood at 7.41%. On average, there were 5.08 coauthors per document and 2901 keywords. Notably, the annual growth rate was calculated at 3.72%.

Publishing Trend of Articles

**Figure 2:**
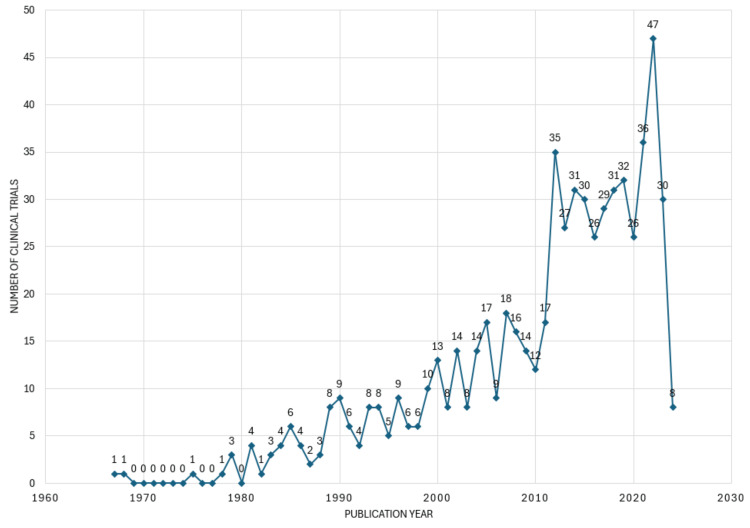
The annual scientific publication of clinical trials on the therapeutic potential of essential oils Credit: Namrata Dagli

The inaugural clinical trial concerning this topic surfaced in the PubMed database back in 1967. Since then, the publication pattern has been sporadic, albeit with a general upward trend. The peak in publication activity was observed in 2022, with the most substantial surge occurring between 2011 and 2012. Conversely, a notable decline was evident between 2022 and 2023. No clinical trials were published between 1969 and 1974; neither were in 1976, 1977, and 1980 (Figure 2).

Most Relevant Authors

**Figure 3:**
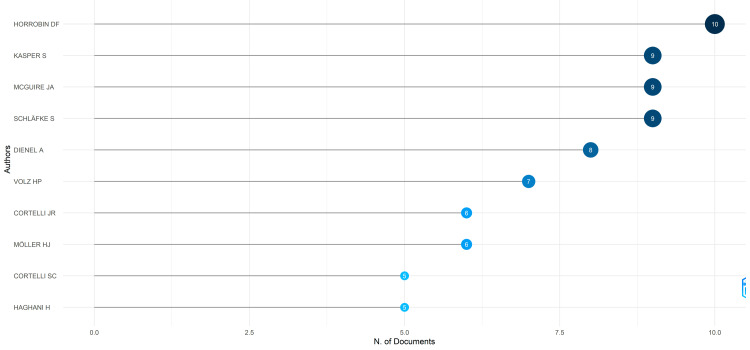
Most relevant authors based on the number of published clinical trials in the PubMed database Credit: Namrata Dagli

The prominence of specific authors, such as Horrobin DF, Kasper S, McGuire JA, and Schlafke S, in published clinical trials on essential oils indicates their significant contributions to this field. Horrobin DF appears to be particularly influential, closely followed by Kasper S, McGuire JA, and Schlafke S. Together, the 10 most relevant authors have collectively contributed to 11.2% of the total published clinical trials in the PubMed database, highlighting their substantial impact and involvement in advancing knowledge and understanding around essential oils through rigorous scientific investigation and dissemination of findings (Figure 3).

Authors' Productivity Analysis

**Figure 4:**
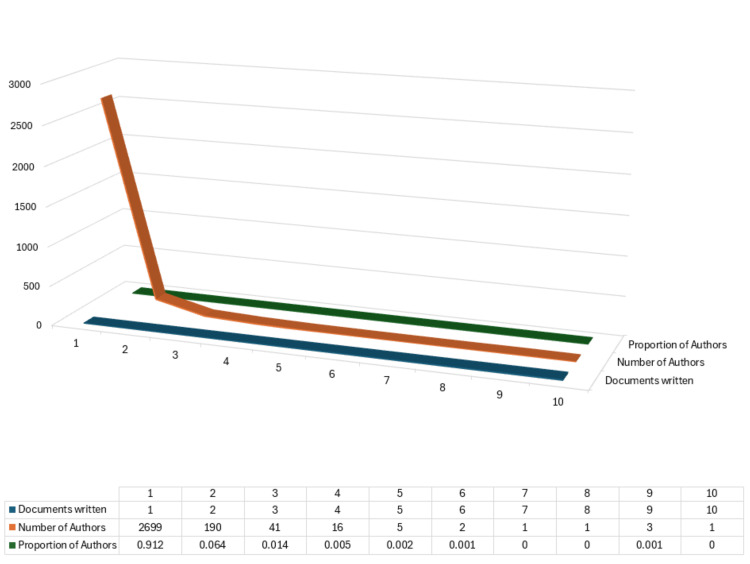
Author’s productivity analysis through Lotka’s Law Credit: Namrata Dagli

Lotka's law, proposed by Alfred J. Lotka, is a principle in bibliometrics that describes the distribution of scientific productivity among authors. Lotka's law states that the number of authors who have published a certain number of papers is inversely proportional to that number. Mathematically, Lotka's law is often expressed using a power-law distribution. It helps to understand the concentration of scientific output among researchers and has implications for research evaluation, collaboration dynamics, and scientific productivity patterns [11]. Figure 4 depicts the authors' productivity in publishing clinical trials. About 91.21% of authors have published only a single paper on the therapeutic potential of essential oils, 6.42% of authors published two papers, and only a few published three or more clinical trials. These findings confirm Lotka's law that there is a small number of highly productive authors, a considerable number of moderately productive authors, and an even more significant number of minimally productive authors (Figure 4).

Coauthorship Analysis of Authors

Three thousand thirty-two authors were identified, each having at least one published clinical trial on the therapeutic potential of essential oils in the PubMed database. Using VOSviewer, the total strength of coauthorship links with other authors was calculated for each author, and those with the highest total link strength (TLS) were chosen for analysis. One thousand authors were included in the overlay visualization. Overlay visualization is a method used to represent authors' coauthorship network graphically. In this visualization, each node represents an author, and the connections between nodes represent collaborations between authors. The size of each node is proportional to the number of documents authored by the author.

**Figure 5:**
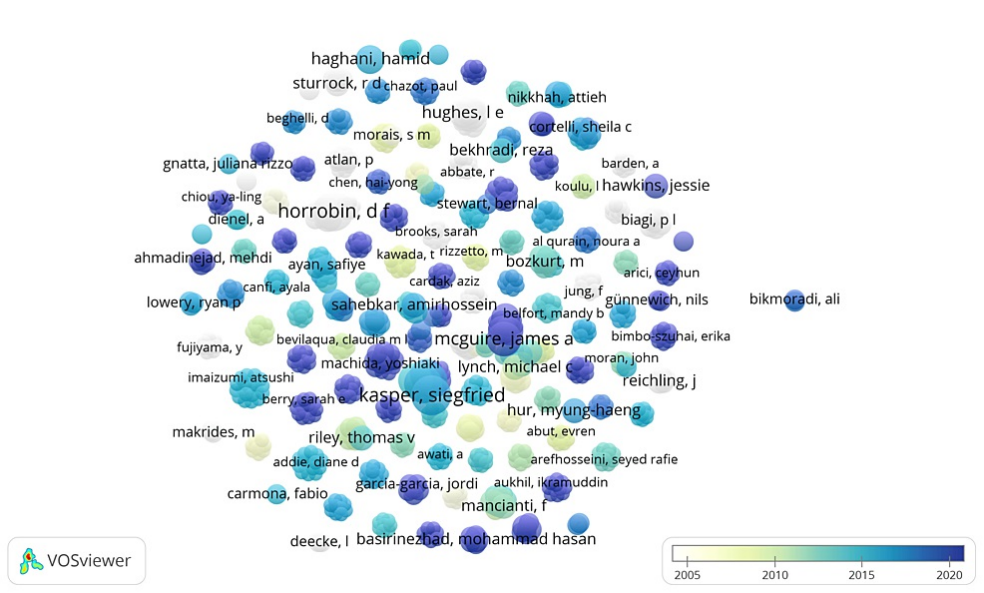
Overlay visualization of coauthorship analysis of authors Weight: Documents, Scores: Average publication year. The size of the circles represents the number of published clinical trials on the therapeutic potential of essential oils. Credit: Namrata Dagli

Additionally, the color of the nodes represents the average publication year of the documents authored by the authors. This visualization helps to understand the structure of collaboration networks and the temporal distribution of the authors' publications. Our analysis revealed 117 clusters of 1000 authors with 4192 links and a TLS of 4567 (Figure 5). Notably, Kasper S had the highest TLS value of 42, followed by Volz HP with a TLS of 33 (Table 1).

**Table 1:** Authors with the highest total link strength values

Authors	Number of published clinical trials	Total link strength
Siegfried Kasper	8	42
Hans-Peter Volz	6	33
James A McGuire	6	31
Hans-Jürgen Möller	5	30
Sandra Schläfke	7	28

Most Relevant Institutions

**Figure 6:**
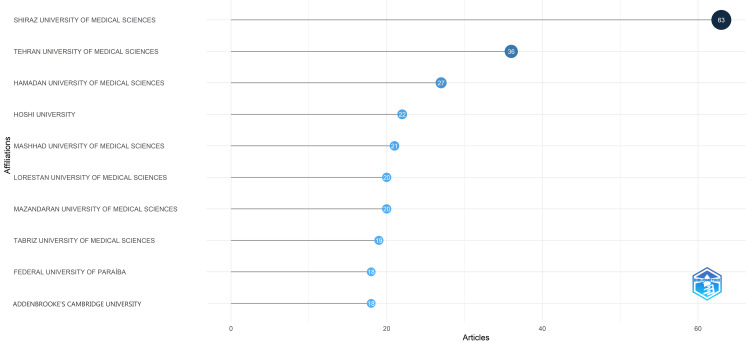
Most relevant institutions based on the number of published clinical trials on the therapeutic potential of essential oils Credit: Namrata Dagli

Figure 6 illustrates the most relevant institutions based on the volume of clinical trials published regarding the therapeutic applications of essential oils. Together, these top 10 institutions accounted for 40% of all publications in the PubMed database concerning the therapeutic potential of essential oils. The Shiraz University of Medical Sciences leads the list, contributing approximately 10% of these publications. The following notable institutions are Tehran University of Medical Sciences, which has 36 publications, and Hamdan University of Medical Sciences, which has 27 publications.

Coauthorship Analysis of Organization

**Figure 7:**
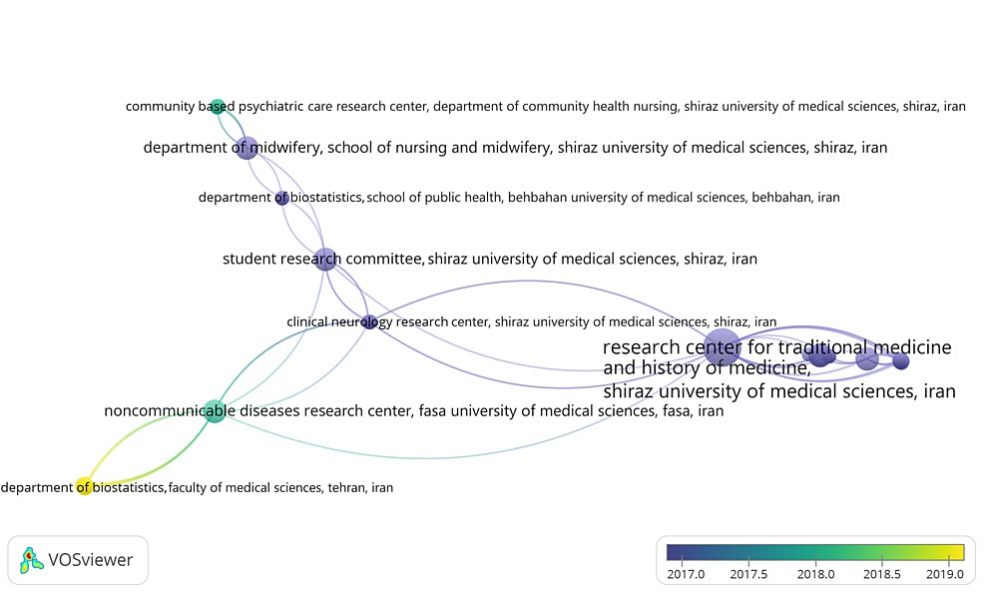
Overlay visualization of institutional coauthorship analysis (weight: documents, scores: average publication year) Credit: Namrata Dagli

One thousand two hundred seventy-four organizations were initially identified, each having at least one published clinical trial on the therapeutic potential of essential oils in the PubMed database. VOSviewer calculated the total strength of coauthorship links between these organizations, and those with the highest TLS were selected for analysis. The most significant connected set comprised 41 items included in the overlay visualization. The overlay visualization of coauthorship analysis of institutions (Figure 7) that assigns weight to "Documents" and scores based on "Average Publication Year" presents a network graph where institutions are nodes, and the connections between them are weighted by the number of documents they have coauthored. The scores are represented by the average publication year, indicating the average year of publication of the documents associated with each institution. In this visualization, institutions are connected by lines, with the thickness of the lines representing the number of documents they have collaborated on.

Additionally, each institution's node is colored based on the average publication year of their papers, providing insights into the temporal distribution of their collaborative research output. The visualization in Figure 7 depicted five clusters of 41 organizations with 171 links and a TLS of 175. The analysis revealed that the organizations with the highest TLS are the Research Center for Traditional Medicine and History of Medicine of Shiraz University of Medical Sciences, with four published clinical trials and 24 TLS, followed closely by the Department of Epidemiology and Biostatistics of Tehran University of Medical Sciences with six published clinical trials and 23 TLS.

Most Relevant Journals

**Figure 8:**
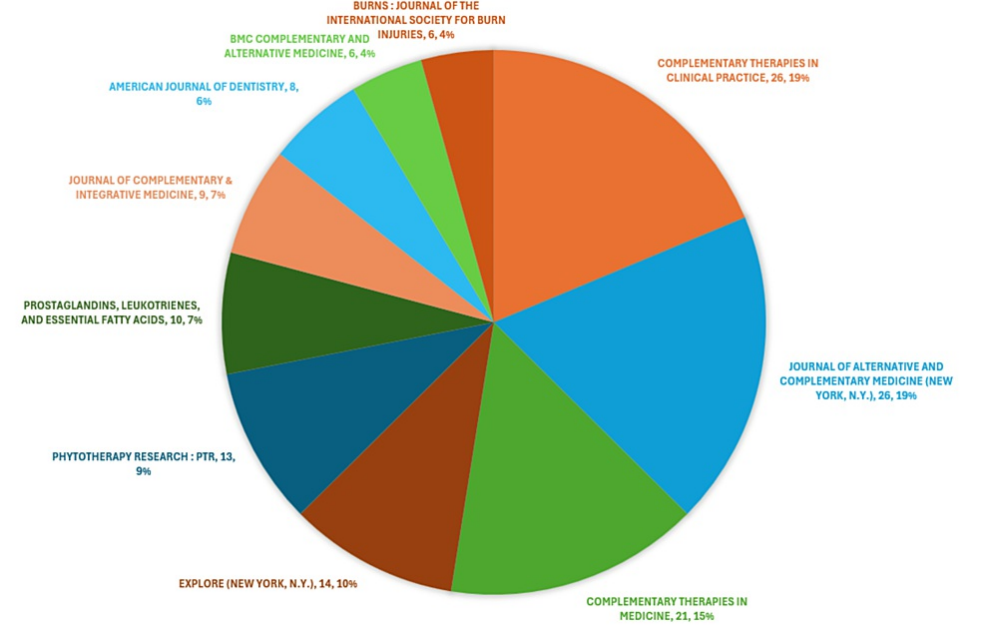
The most relevant journals based on the number of clinical trials published in the PubMed database on the therapeutic potential of essential oils (name of journal, number of published clinical trials, percentage of published clinical trials) Credit: Namrata Dagli

Figure 8 illustrates the primary sources with the highest number of published clinical trials regarding the therapeutic potential of essential oils. Together, these ten most relevant sources account for 21% of all publications in this field. Notably, *Complementary Therapies in Clinical Practice* and the *Journal of Alternative and Complementary Medicine* emerge as the most influential sources, collectively publishing 37.4% of the clinical trials out of the 139 articles published by the most significant journals in this domain.

Keywords Analysis

**Figure 9:**
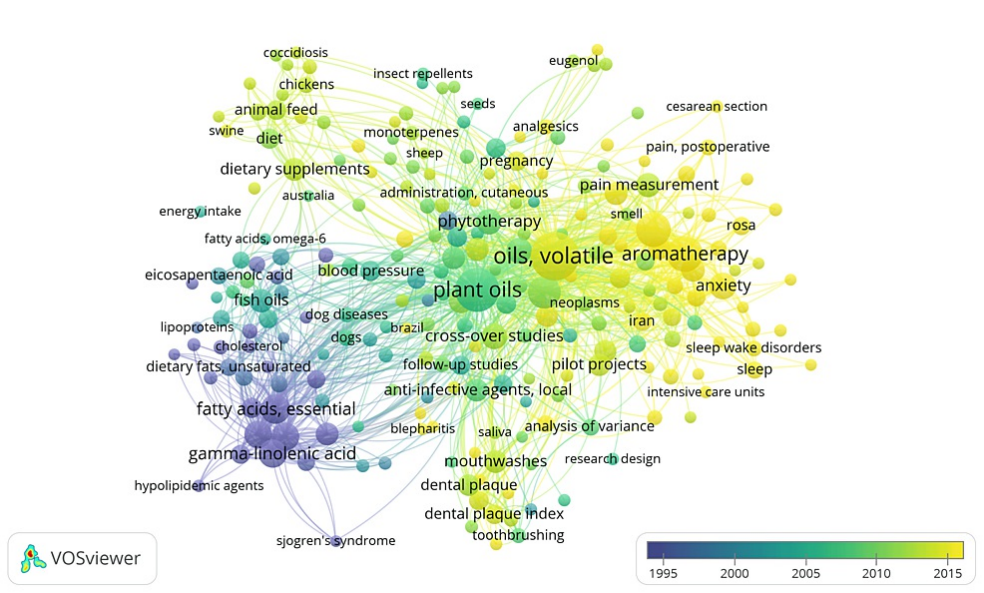
Overlay visualization of co-occurrence of keywords (weight: occurrence, scores: average publication year) Credit: Namrata Dagli

Overlay visualization of keyword co-occurrence, with weight attributed to occurrence and scores based on average publication year, involves illustrating the interrelationships among keywords in a collection of clinical trials focusing on the therapeutic potential of essential oils. Initially, 1724 MeSH keywords were identified with a minimum occurrence threshold of one. Upon adjusting the threshold to five, 251 keywords were identified. After excluding 16 nonspecific keywords such as "human," "animals," and others related to age and gender, the resulting visualization encompasses 235 keywords grouped into seven clusters, revealing 5047 links and 15146. This visualization, depicted in Figure 9, presents the associations between keywords, with the thickness of connections indicating the frequency of their co-occurrence within documents. At the same time, the color denotes the average publication year of those documents. This visualization aids in understanding the patterns of keyword associations and their temporal occurrence within the corpus. Details regarding the items within clusters are provided in Table 2.

**Table 2:** Keywords in clusters identified in co-occurrence analysis

Clusters	MeSH Keywords
Cluster 1	activities of daily living, administration/inhalation, affect, anti-anxiety agents, antineoplastic agents, anxiety, anxiety disorders, aroma therapy, autonomic nervous system, breast neoplasm, citrus, citrus sinensis, combined modality therapy, consciousness, covid-19, critical care dementia, dental anxiety, fatigue, heart rate, depression, intensive care units, Iran, Lavandula, massage, music therapy, neoplasms, nursing homes, odorants, volatile oils, health care outcome assessment, patient satisfaction, pilot projects, post menopause, postoperative nausea and vomiting, psychiatric status rating scales, psychomotor agitation, quality of life, reference values, renal dialysis, research design, the severity of illness index, skin care, sleep, sleep initiation and maintenance disorders, sleep quality, sleep-wake disorders, smell, non-parametric statistics, psychological stress, surveys and questionnaire, treatment outcome, turkey, Zingiber officinale
Cluster 2	acyclic monoterpenes, oral administration, cutaneous administration, animal feed, animal nutritional, physiological phenomena, antibacterial agents, antifungal agents, antioxidant, Australia, body weight, cattle, chicken, coccidiosis, coccidiostats, diet, dietary supplements, digestion, drug dose-response relationship, eimeria, eucalyptus, feces, Foeniculum, gas chromatography-mass spectrometry, insect bites, stings, insect repellents, insecticides, lice infestation, lippia, liver, melaleuca, mice, microbial sensitivity test, monensin, monoterpenes, origanum, phytotherapy, plant extracts, plant leaves, plant preparations, medicinal plants, poultry diseases, Rosmarinus, seeds, sheep, swine, terpenes, thymol, thymus plant, time factors, weight gain
Cluster 3	Anti-inflammatory agents, rheumatoid arthritis, biomarkers, blood pressure, case-control studies, clinical trials, cholesterol, cystic fibrosis, atopic dermatitis, dermatologic agents, dietary fat, unsaturated dietary fats, docosahexaenoic acids, drug administration schedule, eicosapentaenoic acid, emulsion, energy intake, erythrocytes, intravenous fat emulsions, fatty acids, essential fatty acids, omega-3 fatty acids, omega-6 fatty acids, unsaturated fatty acids, fish oils, gamma-linolenic acid, hypertension, hypolipidemic agents, chronic kidney failure, linoleic acids, linolenic acids, lipids, lipoproteins, liver function test, Oenothera biennis, oils, olive oil, parenteral nutrition, phospholipids, plant oils, platelet aggregation, premenstrual syndrome, prostaglandins, random allocation, safflower oil, Sjogren’s syndrome, soybean oil, thromboxane b-2, triglycerides
Cluster 4	Analysis of variance, local anti-infective agents, bacteria, anaerobic bacteria, brain, Brazil, cetyl pyridinium, chronic periodontitis, cognition, microbial colony count, cross-over studies, curcumin, dental plaque, dental plaque index, dental scaling, dentifrices, ethanol, feasibility studies, follow-up studies, gingival hemorrhage, gingivitis, halitosis, longitudinal studies, mouthwashes, oral hygiene, periodontal index, pharmaceutical vehicles, placebos, reproducibility of results, saliva, single-blind method, substance withdrawal syndrome, tooth-brushing, water
Cluster 5	Acne vulgaris, topical administration, ointments, skin, pruritus, analgesics, anti-inflammatory agents, blepharitis, chamomile, chronic disease, diabetic neuropathies, dog diseases, dogs, combination drug therapy, erythema, flavonoids, gels, hydrocortisone, menthol, mite infestations, mites, neuralgia, tea tree oil, turpentine
Cluster 6	Bandages, burns, cesarean section, drug combinations, dry socket, eugenol, iodinated hydrocarbons, pain, pain management, pain measurement, postoperative pain, Para aminobenzoates, postoperative complications, pregnancy, prospective studies, rosa, wound healing
Cluster 7	Breastfeeding, capsules, exercise, dysmenorrhea, irritable bowel syndrome, mentha piperita, parasympatholytics

The keywords in cluster 1 suggest that the research focuses on clinical trials exploring the therapeutic potential of essential oils within the context of complementary and alternative medicine or integrative medicine. These trials likely investigate the use of essential oils in managing a range of health conditions such as anxiety disorders, cancer-related symptoms, sleep disorders, dementia, dental anxiety, postoperative nausea and vomiting, and skin care. Interventions may include aroma therapy, massage, and music therapy using essential oils, focusing on delivery methods and types of essential oils used. Outcomes assessed may include health care outcomes, quality of life, patient satisfaction, and treatment efficacy, measured through psychometric scales, surveys, and physiological parameters such as heart rate and sleep quality. Overall, the research aims to evaluate the effectiveness of essential oils in improving health outcomes and quality of life for various medical conditions. The keywords in cluster 2 suggest research focused on exploring the therapeutic potential of essential oils, particularly in the context of animal health and nutrition. This includes investigating their effects on animal feed, weight gain, digestion, and efficacy against diseases, parasites, and insects. Additionally, studies may examine essential oils' antimicrobial, antioxidant, and pharmacological properties, utilizing clinical trials and chemical analysis techniques to assess their efficacy, safety, and optimal dosage. The keywords in cluster 3 focus on clinical trials investigating the effects of essential oils on inflammation, blood pressure, lipid profiles, platelet aggregation, and other biomarkers associated with conditions such as rheumatoid arthritis, hypertension, and dyslipidemia. The research may involve randomized controlled trials comparing essential oils to standard treatments or placebo, with interventions administered via different routes and formulations. Overall, the studies aim to understand essential oils' efficacy and mechanisms of action in managing various inflammatory and chronic conditions. The keywords in cluster 4 focus on clinical trials investigating the therapeutic potential of essential oils for oral health and periodontal diseases. The research involves assessing the effects of essential oils on factors such as bacteria, dental plaque, gingivitis, and halitosis. Various study designs and methodologies are employed to evaluate efficacy, including analysis of variance, cross-over studies, and longitudinal studies. The trials aim to determine the feasibility and benefits of using essential oils as local anti-infective agents in oral hygiene products like mouthwashes and dentifrices. The keywords in cluster 5 suggest clinical trials exploring the therapeutic potential of essential oils for dermatological conditions. These trials may assess efficacy, safety, mechanisms of action, and comparative effectiveness. They could also investigate chronic disease management. The keywords in cluster 6 suggest that the clinical trials may involve analyzing the efficacy of essential oils in managing pain, particularly postoperative pain. This research could include prospective studies focusing on specific populations like pregnant women, examining potential interactions with conventional medications, and evaluating the efficacy of essential oils such as rose oil. The keywords in cluster 7 indicate clinical trials focused on exploring the impact of essential oils on exercise, breastfeeding, dysmenorrhea, irritable bowel syndrome, and the parasympathetic nervous system. The effectiveness and safety of ingesting them in capsule form. The trials were centered on specific essential oils like Mentha piperita.

Word Cloud

**Figure 10:**
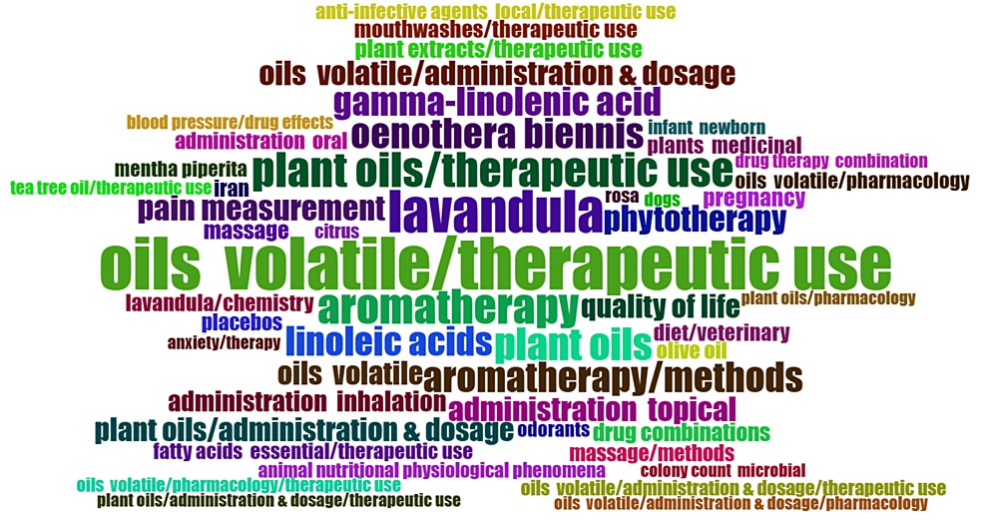
Word map showing the most frequently used keywords (word occurrence: square root of frequency) Credit: Namrata Dagli

A word cloud is a visual representation of text data, where the size of each word corresponds to its frequency or importance within the dataset. A word cloud provides a quick visual summary of the most prominent themes or topics within the dataset, helping researchers identify recurring terms and gain insights into the focus areas of the clinical trials on essential oils. In Figure 10, a word cloud displays vital terms or phrases related to the clinical trials on the therapeutic potential of essential oils. Words that appear more frequently in the dataset are represented with larger font sizes, making them more prominent in the visualization. To enhance the relevance of the terms displayed, nonspecific words, including "human," "animal," and other age and gender-related terms, were removed. According to the word cloud (Figure 10), the 10 most frequently used terms are "therapeutic use of volatile oils," "Lavandula," "aromatherapy," "therapeutic uses of plant oils," "Oenothera biennis," "Gamma-linolenic acid," "linoleic acid," "phytotherapy," "quality of life," and "pain measurement."

Analysis of Topic Trends

The analysis of topic trends across different periods reveals notable shifts in the focus of clinical trials on the therapeutic potential of essential oils.

During the period spanning from 1985 to 1996, the emphasis of clinical trials was primarily on investigating the therapeutic properties of plant oils, particularly Oenothera biennis, and essential fatty acids such as linolenic acid and linoleic acid. This era marked a foundational exploration into the potential health benefits of essential oil constituents and their impact on various health conditions.

Between 1997 and 2009, there was a shift in research focus toward broader topics such as medicinal plants beyond essential oils, including olive oil and chamomile, placebo effects, and investigating different routes of administration for these substances. Notably, there was an increased interest in studying the impact of essential oils on children, particularly preschool-aged children, indicating a growing awareness of pediatric health considerations. Additionally, canine subjects became prominent in this period, likely reflecting research into veterinary applications of essential oils. The prevalent study design during this time was predominantly double-blind follow-up studies, reflecting a rigorous approach to scientific inquiry.

**Figure 11:**
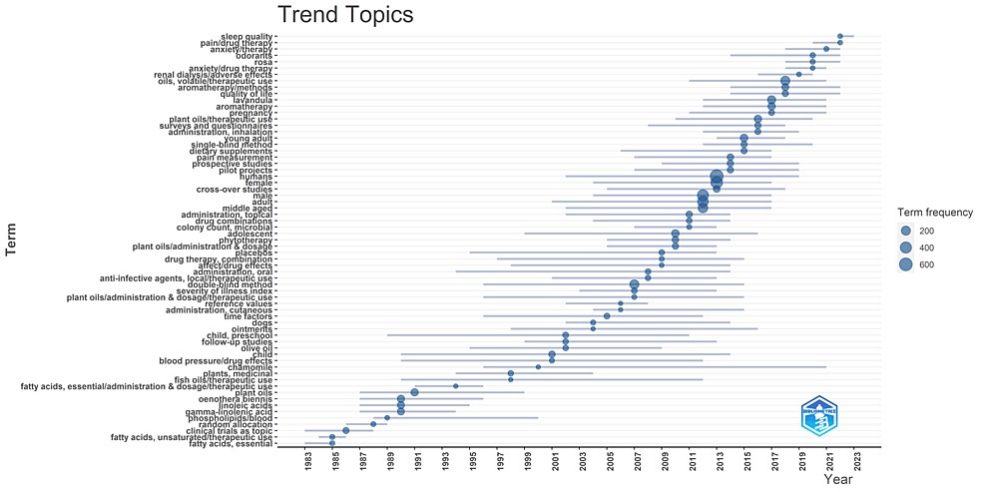
Analysis of topic trends of clinical trials on essential oils published in the PubMed database Credit: Namrata Dagli

From 2010 to 2023, clinical trials began to explore new avenues of research, including the effects of essential oils on sleep quality, pain management, anxiety reduction, and the use of odorants and aromatherapy in various contexts. Moreover, there was a notable focus on evaluating the impact of essential oils on quality of life, pregnancy-related concerns, dietary supplements, and phytotherapy. Interestingly, the study population shifted toward mainly including young adults and adolescents during this period, reflecting a broader demographic range of interest. Furthermore, the observation that "female" was more frequently used than "male" suggests an increased focus on gender-specific considerations in research studies. The predominant study designs shifted toward prospective and single-blinded trials. Certain plant species, notably rosa and lavandula, emerged as the most studied botanical sources of essential oils, reflecting their widespread use and therapeutic potential. Overall, the trends observed highlight the dynamic nature of research on essential oils (Figure 11).

Thematic Evolution

**Figure 12:**
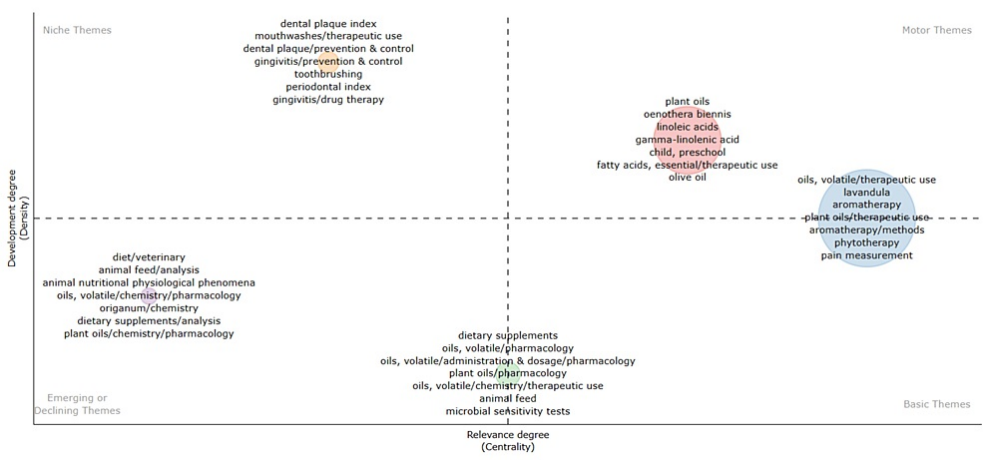
Thematic evolution analysis Weight: Word occurrences; Clustering algorithm: Walktrap; Minimum weight index: 0.1; Minimum cluster frequency per thousand documents: 10 Credit: Namrata Dagli

The thematic evolution depicted in Figure 12 illustrates a distinct progression in the focus of clinical trials on essential oils over time. From the early years until 2007, research primarily centered around investigating the Oenothera Biennis plant, animal nutritional and physiological phenomena, therapeutic uses of volatile oils, patch tests, and therapy of acne vulgaris. This period explores the potential therapeutic benefits of specific plant extracts and their applications in addressing dermatological concerns and nutritional aspects in animals.

Between 2008 and 2017, there was a noticeable shift toward broader themes such as phytotherapy, tea tree oil, adolescents' nutritional and physiological phenomena, weight gain, and diet. This era saw increased attention to phytotherapeutic approaches and the exploration of tea tree oil's potential therapeutic applications. Furthermore, studies began to include infants, indicating a widening scope of demographic considerations.

After 2017, the focus of research shifted toward investigating origanum, tea tree oil, and olive oil, with a particular emphasis on exploring routes of administration, effects on blood pressure, and cognition. Additionally, their therapeutic use in mouthwashes and combination therapy was investigated. This period reflects a trend toward more targeted investigations into specific essential oils and their diverse applications in various health contexts.

Comparing these findings with the analysis of topic trends mentioned earlier, there are notable similarities in the overarching themes and shifts observed over time. Both studies highlight a progression from foundational explorations of essential oil constituents and safety testing toward broader applications and more targeted investigations into specific oils and therapeutic areas. Additionally, there is a parallel in including demographic considerations such as pediatric and animal subjects across different periods. However, differences exist in the topics and oils studied, indicating the need for various types of analysis for a holistic understanding of the research topic.

Thematic Map

**Figure 13:**
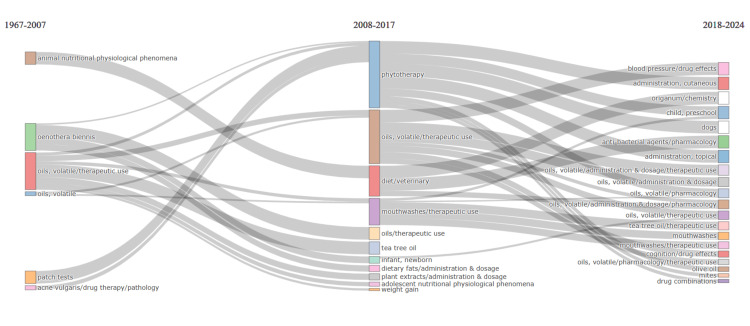
Thematic Map based on clinical trials published in the PubMed database on the therapeutic potential of essential oils Credit: Namrata Dagli

Figure 13 serves as a thematic map, highlighting spatial patterns of themes relevant to the therapeutic potential of essential oils and categorizing them into four categories based on their relevance and development: motor, fundamental, niche, and emerging or declining. The X-axis denotes the degree of relevance, while the Y-axis represents the degree of development. Notably, relevant themes encompass therapeutic uses of volatile oils, Lavandula, aromatherapy, phytotherapy, and pain measurement. Themes slightly less relevant but more developed include Oenothera Biennis, linoleic acids, gamma-linolenic acid, essential fatty acid, and olive oils. Conversely, the most developed themes not pertinent to the topic are related to dentistry, such as dental plaque index, therapeutic uses in mouthwashes, dental plaque, gingivitis, tooth brushing, and periodontal index. Additionally, emerging or declining themes involve studies of essential oils in animals.

Analysis of Countries of Corresponding Authors and Their Collaboration Frequency

**Figure 14:**
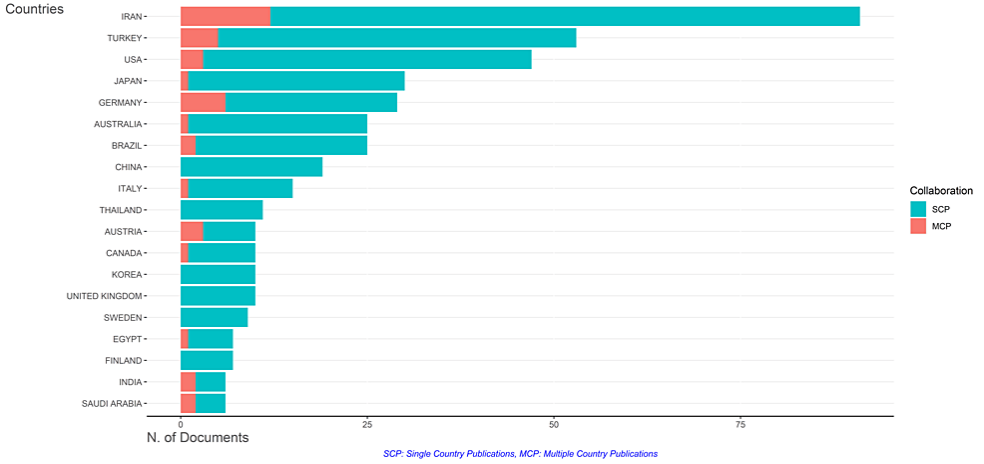
Most relevant countries based on the number of published clinical trials in the PubMed database and their collaboration pattern Credit: Namrata Dagli

Figure 14 underscores that most published clinical trials are conducted within a single country. The data from the figure indicates that Iran exhibits the highest frequency of collaboration, closely followed by Germany and Turkey. Notably, China, Korea, the United Kingdom, Sweden, and Finland did not collaborate with other countries. On the other hand, Japan, Australia, Italy, Canada, and Egypt demonstrated minimal collaboration. India, Saudi Arabia, and Brazil exhibited a similar collaboration frequency, slightly higher than the minimum.

**Figure 15:**
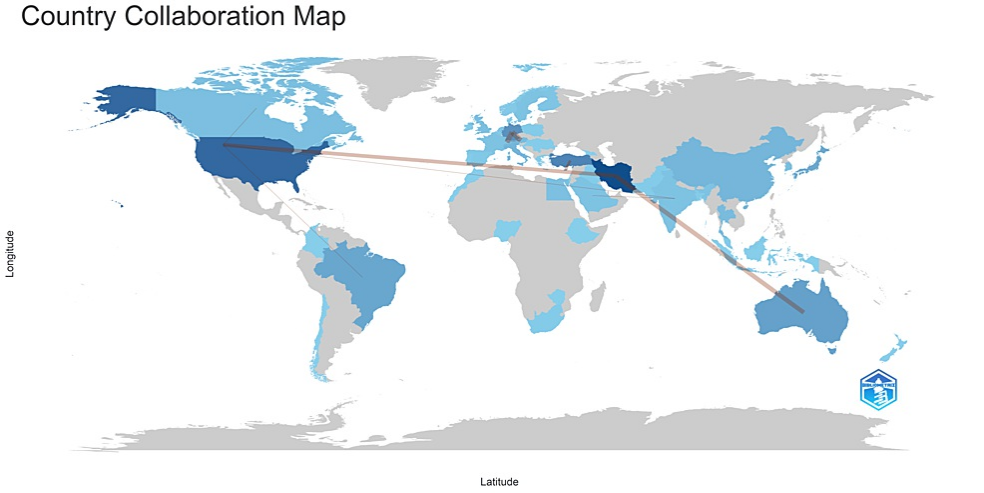
Collaboration between various countries across the world Credit: Namrata Dagli

Figure 15 illustrates the collaboration between different countries in the context of published clinical trials. The intensity of the blue color reflects the number of clinical trials conducted by each country, providing insight into their research output in this field. Additionally, the thickness of the connecting lines signifies the frequency of collaboration between the countries they link. According to the figure, the highest frequency of collaboration is observed between Germany and Austria, as well as between Germany and Switzerland, indicating strong research partnerships in clinical trial endeavors. Following closely behind is the collaboration between Iran and Australia, suggesting active engagement and cooperation in conducting clinical research across borders. Overall, this visualization offers valuable information on the extent and nature of international collaboration in clinical trials, highlighting key partnerships and areas of active engagement among countries in advancing scientific knowledge and medical research.

Discussion

Clinical trials on the topic exhibited an irregular publishing pattern with an overall increasing trend, peaking in 2022. Notable fluctuations were observed, including a significant rise between 2011 and 2012 and a pronounced decline between 2022 and 2023, with gaps in publication years noted between 1969-1974 and 1976, 1977, and 1980. The irregular pattern of publishing clinical trials on essential oils could stem from various factors, including shifts in research funding, evolving scientific interest, regulatory changes affecting research priorities, emerging trends in alternative medicine, fluctuations in demand from the healthcare industry, and advancements in technology facilitating research methodologies. Additionally, variations in the availability of essential oil-related products, potential controversies or debates within the scientific community, and the complexity of conducting clinical trials on natural substances could also contribute to the observed publishing pattern.

Six hundred and sixty-one articles were selected for analysis, authored by 2959 individuals, and published across 359 distinct sources. Noteworthy is the influence of key authors like Horrobin DF, Kasper S, McGuire JA, and Schlafke S. Lotka's law underscores the distribution of authors' productivity, revealing a small number of highly productive authors. Coauthorship analysis identifies significant collaborations among authors and institutions, with prominent contributors like Siegfried Kasper and institutions like Shiraz University of Medical Sciences. Furthermore, the study highlights leading journals like *Complementary Therapies in Clinical Practice* and the *Journal of Alternative and Complementary Medicine*, shaping the discourse in this domain.

The clusters of keywords indicate a diverse range of research areas exploring the therapeutic potential of essential oils through clinical trials. These trials explore their efficacy in complementary medicine for conditions like anxiety and cancer symptoms, as well as in animal health and nutrition, dermatological care, and pain management, including postoperative pain. Other trials focus on oral health, inflammation, and conditions like hypertension. They assess various outcomes, such as quality of life and safety, employing techniques like aroma therapy and massage. Additionally, studies examine essential oils' impacts on exercise, breastfeeding, and gastrointestinal disorders, aiming to understand their effectiveness and safety in capsule form and specific oil types like Mentha piperita. Thematic maps categorize themes based on relevance and development, highlighting prominent areas of study and emerging trends. The analysis of topic trends reveals shifting trends in clinical trials on essential oils over three distinct periods, with initial emphasis on investigating plant oils and essential fatty acids, followed by broader research into various applications, including pediatric and veterinary use, and culminating in explorations of novel therapeutic avenues, demographic shifts, and gender-specific considerations, underscoring the dynamic evolution of research in the field. The thematic evolution of clinical trials on essential oils reveals a transition from early explorations of specific plants and dermatological concerns to broader investigations into phytotherapy and specific oils, with a notable inclusion of demographic considerations such as adolescents, infants, and animals, reflecting a dynamic and evolving research landscape over time. Thematic map categorizes themes related to the therapeutic potential of essential oils based on their relevance and development, highlighting notable topics such as aromatherapy and phytotherapy alongside less pertinent but more developed themes like dental care while identifying emerging or declining research areas such as animal studies. Analysis of countries of corresponding authors suggests that Iran has the highest number of multiple-country publications. Additionally, analyses of country collaborations reveal patterns of international research partnerships, with certain countries demonstrating higher levels than others.

No bibliometric studies were found on this topic. Most similar studies are focused on aroma therapy [12,13] and antimicrobial effects [14-16]. Moreover, several studies were solely dedicated to examining the properties of individual essential oils [17,18]. Additional bibliometric analyses were discovered focusing on essential oils but with alternative objectives unrelated to therapy. These analyses explored areas such as their utilization as biofuel, their applications in agriculture, their biocompatibility with fungi, the process of nanoencapsulation, and their efficacy in various contexts [19-24].

**Figure 16:**
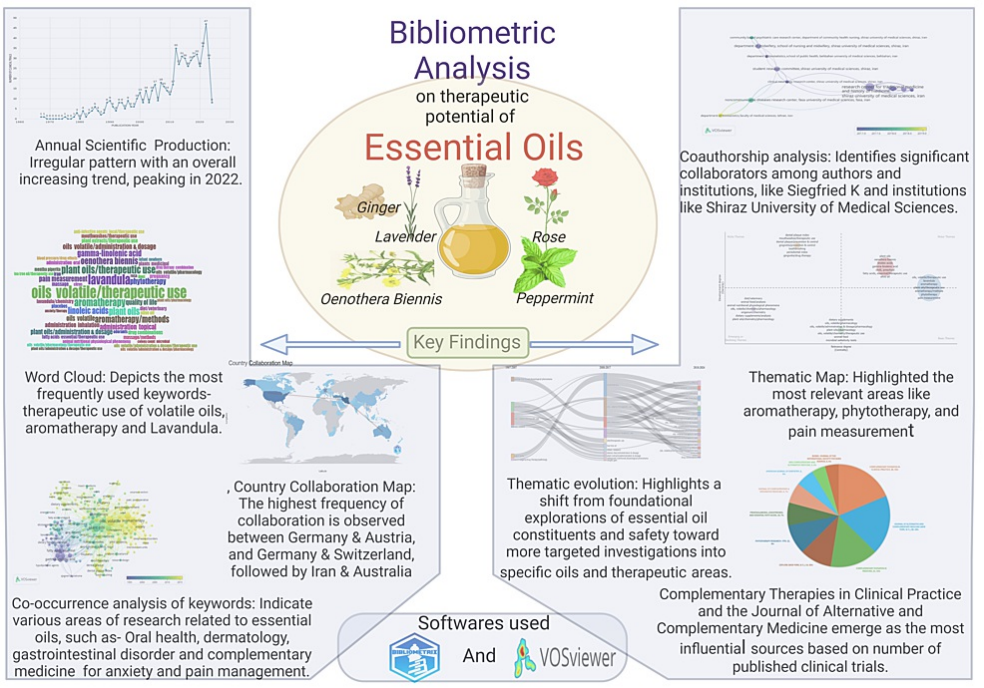
Key findings of the bibliometric analysis on the therapeutic potential of essential oils This figure has been drawn with the premium version of BioRender [25] (https://biorender.com/, accessed on March 19, 2024) with the Agreement License Number ZT26LG5ML0. Credit: Namrata Dagli

The study's main limitation is that the conclusion is based on the data obtained from only one database. Another limitation is that the investigation is quantitative, and the quality of individual papers was not analyzed. Regardless of these limitations, this study offers a holistic understanding of the research trends, thematic shifts, and collaborative efforts shaping the exploration of essential oils' therapeutic potential in clinical trials. Also, it would help identify the leading researchers and organizations for budding researchers. The key findings of this analysis are summarized in Figure 16.

﻿Future study recommendations

Future research on the therapeutic potential of essential oils should address several key areas identified through bibliometric analysis and thematic trends. First, there is a need for more comprehensive investigations into the efficacy of essential oils in clinical trials, focusing on diverse applications such as complementary medicine for conditions like anxiety, cancer symptoms, and pain management, alongside areas like oral health, inflammation, and gastrointestinal disorders. These trials should prioritize assessing outcomes such as quality of life, safety, and specific therapeutic mechanisms, utilizing techniques like aroma therapy and massage while considering demographic factors such as age and gender. Second, future studies should aim to overcome the limitations of existing research by conducting high-quality, multicenter trials with standardized methodologies and rigorous quality control measures to ensure the reliability and generalizability of findings. Additionally, researchers should try to understand the reasons behind the increase and decrease in publications. Finally, there is a need for more focused bibliometric analyses to track evolving research trends, identify emerging areas of interest, and inform strategic research priorities in the field of essential oils and their therapeutic applications.

## Conclusions

Clinical trials on the therapeutic potential of essential oils demonstrated an irregular publication pattern, showing a general upward trend with a peak in 2022, characterized by notable fluctuations, notably a substantial increase from 2011 to 2012 and a marked decrease from 2022 to 2023. The analysis identified Horrobin DF as the most influential author in this domain, closely followed by Kasper S, McGuire JA, and Schlafke S. Lotka's law emphasizes the distribution of author productivity, indicating a minority of highly productive authors. Through coauthorship analysis, substantial collaborations among authors and institutions emerge, with notable contributors such as Siegfried Kasper and establishments like Shiraz University of Medical Sciences. Additionally, the analysis underscores prominent journals such as *Complementary Therapies in Clinical Practice* and the *Journal of Alternative and Complementary Medicine*. Through keyword clustering, associations between different topics and their temporal occurrence are elucidated, shedding light on the evolving research landscape. Also, it unveiled the diverse applications of essential oils across dermatology, dentistry, oral health, mental well-being, and pain and anxiety management. The analysis of topic trends and thematic evolution reveal shifts in research focus over time, highlighting a progression from foundational explorations of essential oil constituents toward broader applications and more targeted investigations into specific oils and therapeutic areas.

Analysis of the countries of corresponding authors suggests that Iran has the highest number of multiple-country publications. Additionally, collaboration analysis among countries highlights Germany and Switzerland as well as Germany and Austria as leading collaborators, with notable partnerships observed between Iran and Australia. Overall, the analyses provide comprehensive insights into keyword associations, thematic evolution, and international collaboration in essential oil research, offering valuable perspectives for understanding trends and priorities in this field.
